# Determinants of Arterial Stiffness in Patients with Morbid Obesity. The Role of Echocardiography and Carotid Ultrasound Imaging

**DOI:** 10.3390/medicina59030428

**Published:** 2023-02-22

**Authors:** Viviana Aursulesei Onofrei, Carmen Lacramioara Zamfir, Ecaterina Anisie, Alexandr Ceasovschih, Mihai Constantin, Florin Mitu, Cristina Andreea Adam, Elena-Daniela Grigorescu, Antoneta Dacia Petroaie, Daniel Timofte

**Affiliations:** 1 Department of Medical Specialties I and II, Morpho-Functional Sciences I and Preventive Medicine and Interdisciplinary, “Grigore T. Popa” University of Medicine and Pharmacy, University Street No. 16, 700115 Iasi, Romania; 2 “St. Spiridon” Clinical Emergency Hospital, Independence Boulevard No. 1, 700111 Iasi, Romania; 3 Clinical Rehabilitation Hospital, Cardiovascular Rehabilitation Clinic, Pantelimon Halipa Street No. 14, 700661 Iasi, Romania; 4 Academy of Medical Sciences, Ion C. Brătianu Boulevard No 1, 030167 Bucharest, Romania; 5 Academy of Romanian Scientists, Professor Dr. Doc. Dimitrie Mangeron Boulevard No. 433, 700050 Iasi, Romania

**Keywords:** arterial stiffness, cardiovascular risk, morbid obesity, pulse wave analysis, adiponectin, chemerin, adipocyte

## Abstract

*Background and objective:* Morbid obesity is accompanied by an increased cardiovascular (CV) risk, which justifies a multidisciplinary, integrative approach. Arterial stiffness has a well-defined additional role in refining individual CV risk. Given that echocardiography and carotid ultrasound are usual methods for CV risk characterization, we aimed to identify the imaging parameters with a predictive value for early-onset arterial stiffness. *Material and methods:* We conducted a study in which 50 patients (divided into two equal groups with morbid obesity and without obesity), age and gender matched, untreated for cardiovascular risk factors, were addressed to bariatric surgery or non-inflammatory benign pathology surgery. Before the surgical procedures, we evaluated demographics, anthropometric data and biochemical parameters including adipokines (chemerin, adiponectin). Arterial stiffness was evaluated using the Medexpert ArteriographTM TL2 device. Transthoracic echocardiography and carotid ultrasound were also performed. We also analyzed adipocyte size and vascular wall thickness in intraoperative biopsies. *Results:* Left ventricle (LV) mass index (*p* = 0.2851), LV ejection fraction (LVEF) (*p* = 0.0073), epicardial adipose tissue thickness (*p* = 0.0001) as echocardiographic parameters and carotid intima–media thickness (*p* = 0.0033), relative wall thickness (*p* = 0.0295), wall to lumen thickness ratio (*p* = 0.0930) and carotid cross-sectional area (*p* = 0.0042) as ultrasound parameters were significant measures in our groups and were assessed in relation to adipocyte size, blood vessel wall thickness and adipokines serum levels. Statistical analysis revealed directly proportional relationships between LV mass index (*p* = 0.008), carotid systolic thickness of the media (*p* = 0.009), diastolic thickness of the media (*p* = 0.007), cross-sectional area (*p* = 0.001) and blood vessel wall thickness. Carotid relative wall thickness positively correlates with adipocyte size (*p* = 0.023). In patients with morbid obesity, chemerin and adiponectin/chemerin ratio positively correlates with carotid intima–media thickness (*p* = 0.050), systolic thickness of the media (*p* = 0.015) and diastolic thickness of the media (*p* = 0.001). The multiple linear regression models revealed the role of epicardial adipose tissue thickness and carotid cross-sectional area in predicting adipocyte size which in turn is an independent factor for arterial stiffness parameters such as pulse wave velocity, subendocardial viability ratio and aortic augmentation index. *Conclusions:* Our results suggest that epicardial adipose tissue thickness, carotid intima–media thickness, relative wall thickness and carotid cross-sectional area might be useful imaging parameters for early prediction of arterial stiffness in patients with morbid obesity.

## 1. The role of Imaging Methods in Cardiovascular Risk Assessment

The assessment of global cardiovascular risk as well as the risk of a potentially fatal acute cardiovascular event is a permanent concern of the academic community, and research on this topic is constantly being reported, both for therapeutic and prognostic purposes [1]. According to the World Health Organization reports, cardiovascular disease (CVD) is one of the leading causes of death worldwide, accounting for approximately 18 million deaths annually [2,3]. The negative impact of industrialization on global technology is reflected in the increasing prevalence of obesity in all age groups, one of the main cardiovascular risk factors [4]. Its association with hypertension, diabetes, dyslipidemia or environmental factors (intensively researched in recent years in relation to the risk of cardiovascular morbidity and mortality) requires the development of multidisciplinary prevention and treatment strategies [5,6]. 

The integration of imaging diagnostic methods and the dosage of molecules with a predictive role on the risk of an acute cardiovascular event are useful tools in clinical practice and research alike, and the evidence of correlations between them opens new research directions [7]. The connection between arterial stiffness and obesity has been highlighted in multiple clinical studies in the literature. In patients with obesity, excessive secretion of adipokines leads to the maintenance of pro-inflammatory status and to the appearance and subsequent development of atherosclerosis [8]. 

Adiponectin and chemerin are two of the studied cytokines in relation to arterial stiffness, and they also correlate with a number of imaging methods or functional markers [9,10]. Thus, echocardiography and carotid Doppler ultrasound are two basic imaging investigations that should be performed in patients with obesity. Clinical studies in the literature have shown links between these methods, inflammatory biomarkers, excess adipokines secreted by visceral adiposity and arterial stiffness parameters. Thus, multidisciplinary imaging assessment highlights predictors of the arterial stiffness process, thus contributing to the individualization of the associated cardiovascular risk.

The aim of this study was to evaluate echocardiographic and Doppler ultrasound carotid parameters in order to identify predictors for arterial stiffness with a therapeutic and prognostic role in patients with morbid obesity.

## 2. Materials and Methods

### 2.1. Study Design and Population

**Figure 1:**
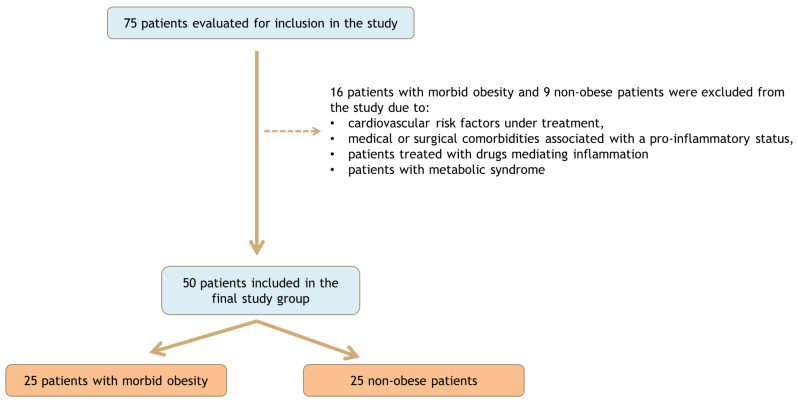
Flow chart of the studied group.

We conducted a case–control study which included 50 adult patients divided into two groups as follows: the first group with 25 consecutive patients with morbid obesity (defined as a body mass index (BMI) above 40 kg/m^2^) and the second group with 25 age and gender-matched non-obese patients (with an BMI less than 30 kg/m^2^) (Figure 1). The study period was January–May 2017. All patients underwent laparoscopic surgical procedures in the 3rd General Surgery Department of “St. Spiridon” Hospital. Patients with morbid obesity underwent bariatric surgery, and those in the second group underwent non-inflammatory benign pathology surgery. All patients underwent a thorough cardiological check-up prior to surgery. The exclusion criteria were represented by patients with cardiovascular risk factors under treatment, medical or surgical comorbidities associated with a pro-inflammatory status, or treated with drugs mediating inflammation. In addition, we excluded patients with 3 of 5 of the criteria for metabolic syndrome [11,12] or those participating at the same time in another clinical trial. Patients included in the study had no associated cardiovascular risk factors requiring drug treatment.

### 2.2. Laboratory Measurements 

We evaluated demographics (age, gender), cardiovascular (CV) risk factors, anthropometric parameters, hemodynamics (systolic and diastolic blood pressure (SBP, DBP [mmHg]), biochemical and hematological data as well as arterial stiffness (AS) parameters. Anthropometric measurements included BMI (kg/m^2^), waist circumference, waist to hip circumference ratio (WHR) and index of central obesity [13] defined as waist circumference to height ratio.

The assessment of biochemical parameters was performed within two hours. Fasting glucose, total cholesterol and triglycerides were determined applying the enzymatic colorimetric method, while high-density lipoprotein (HDL) cholesterol was measured using imunoturbidimetry. Low-density lipoprotein (LDL) cholesterol levels were calculated by the Friedewald equation [14]. Fasting insulinemia and serum TNF-α—as marker of inflammation were assessed by chemiluminescence immunoassay kits (Siemens Healthcare GmbH., Germany) automated by an IMMULITE 1000 analyzer. Insulin resistance (IR) and insulin sensitivity (IS) completed the assessment of metabolic profile and were calculated by applying the Homeostasis Model Assessment (HOMA) and quantitative check index (QUICKI) [15]. 

Serum adipokines (chemerin and adiponectin) were processed after venous blood collection in vacutainer tubes without anticoagulant and centrifugation. Chemerin, known as retinoic acid receptor responder protein 2—RARRES2, is a 14 kDa protein, 131–137 amino acids long, resulted from proteolytic cleavage of the inactive molecule [16]. Serum chemerin and adiponectin were assessed by quantitative specific Human ELISA (enzyme-linked immunosorbent assay) kits (ab155430 and ab99968, respectively) supplied by Abcam Cambridge, U.K., for research use only. The chemerin/adiponectin ratio was calculated for studying the relation with AS [17]. All biological samples were stored at −20 °C and processed after completing the enrollment of patients [15,18].

### 2.3. Arterial Stiffness Evaluation

Arterial stiffness was assessed by oscillometric method using the Medexpert ArteriographTM TL2 device. Data entry into the software (identification data, anthropometric parameters, age) and brachial blood pressure (BP) measurement generated the AS parameters: aortic pulse wave velocity (PWV), aortic and brachial augmentation index (Aix), central BP, central pulse pressure (PP) and subendocardial viability index (SEVR); this was assessed according to the 2012 Expert consensus document [19]. By this method, a complete report is produced in about 10 min.

The measurement protocol via the Arteriograph device involved the following steps: (1) recording general patient data (name, date of birth, weight, height, arm circumference and abdominal circumference, the distance between the sternal notch and the upper edge of the pubic symphysis, without following the abdominal relief); (2) locating the area of maximum pulsatility of the brachial artery, with the positioning of the cuff of the device at this level; (3) the initiation of measurements, with the patient lying on his back and tracking the recording of pulse waves on the monitor to observe the morphology of the route; and (4) the interpretation of the results.

Patients received dietary recommendations prior to the evaluation; smoking and coffee consumption were prohibited 3 h prior to the investigation. Nitrates and alcohol consumption were prohibited 10 h before [15].

### 2.4. Transthoracic Echocardiography and Carotid Doppler Ultrasound

Transthoracic echocardiography was part of the assessment prior to surgery and was performed using Ecograph MyLabTM 30Gold Cardiovascular (ESAOTE-SPA ITALIA). Studied parameters were obtained by applying classical echocardiographic techniques according to the European Association of Cardiovascular Imaging recommendations [20]. M-mode, 2D assessment, continuous and pulsed Doppler assessment were used to obtain morphological and functional parameters. Ejection fraction was calculated using Simpson biplane method. Epicardial adipose tissue thickness, a component of the visceral adiposity, was measured between the epicardium of the right ventricle and parietal pericardium through parasternal long and short axis windows [21,22].

Carotid Doppler ultrasound was performed in all patients enrolled in the study, with an appropriate probe of the same ultrasound scanner. Carotid intima–media thickness, systolic thickness of the media, diastolic thickness of the media, relative wall thickness, wall-to-lumen thickness ratio and carotid cross-sectional area were the main parameters assessed [23,24]. All parameters were assessed according to current clinical guidelines [25]. Carotid intima–media thickness was measured using the edge–to–leading edge technique for measuring the blood–intima and media–adventitia interfaces of the far wall.

All echocardiographic measurements were carried out with an Esaote MyLab 30 Gold CV cardiovascular ultrasound system (Esaote, Geneva, Italy) equipped with a 3.5 MHz phased-array transducer (PA230) and a 7–12 Mhz linear array transducer (LA523). The used frame rates were 28–30 per second for echocardiography and 35 per second for carotid ultrasound. Measurements were obtained independently by two blinded cardiologists that included a set of predefined parameters. All ultrasound images obtained by the two cardiologists were stored in digital format and analyzed independently by two blinded investigators to assess the interpretability of the images using a standardized rating scale. The evaluation of pulse-wave velocity was performed by only one cardiologist with expertise in these measurements.

### 2.5. Intraoperative Biopsy for Local Adipocytes Evaluation

During surgery procedures, 1 cm^3^ specimens from the great/small epiploon region, including a visible small artery for local adipocytes assessment, were collected. Histological samples were formalin-fixed paraffin embedded (FFPE), offering significant morphological details of white adipose tissue. We preferred FFPE to cryosectioning because multiple sections were required, so sectioning of paraffin blocks using microtome (5 μm/slice thickness) allowed us to obtain sufficient sections for microscopic exam. H&E staining was used as it allows the visualization of adipose tissue morphology and the changes in tissular or individual cell expansion. A blinded manner was used in the microscopic exam and the magnification was x200 and x400. We used Zeiss Observer Z1 Tissue Gnostics 9 Tissue Facks software in order to perform scanning and analysis for each slide. Adipocyte size was examined on 5 different fields on the prepared slides and the results were compared using a qualitative scale. Initially, the long axis of the adipocytes was determined in order to make an assessment of the order of cells size in the two groups. The adipocytes at the centre and four extremities of each image were morphologically characterized. The vascular wall thickness was also measured. The fields selected for analysis were chosen from the four extremities of each histopathological image so as to include vessels with the most different caliber and wall profile.

### 2.6. Statistical Analysis

Statistical analysis was performed using SPSS statistics software (Statistical Package for the Social Science version 20, for Windows; SPSS Inc., Chicago, IL, USA). For the continuous type variables, the mean, median, minimum and maximum values, quartiles and standard deviation were calculated. Skewness (measuring the symmetry of the variables with respect to the mean value) and kurtosis (flattening coefficient) were determined to assess the normal distribution of continuous variables.

To compare the mean values between two groups of continuous values in order to determine the statistically significant differences, the *t* test (independent *t* test) and ANOVA (one-way analysis of variance) were used. Pearson and Spearman (r) correlation coefficients were used to assess the presence of correlations between the studied variables. A natural logarithmic transformation was performed for the variables without a normal distribution. Kendall tau coefficient was used to determine the same correlations for the whole sample. For the subsequent analysis of the relationship between the variables that met the statistical threshold for the realized correlations, a simple linear regression was performed and also by selecting several independent variables that influence a dependent variable, simple linear regression was extended to multiple regression. Multiple linear regression models were created to analyze the risk factors that determine the arterial stiffness for the obese group a *p*-value < 0.05 was considered statistically significant.

### 2.7. Ethics

The study protocol was approved by the local Ethics Committees of “Grigore T. Popa” University of Medicine and Pharmacy Iasi and of “St. Spiridon” Clinical Emergency Hospital Iasi, and was conducted in accordance with the terms of the Helsinki Declaration. All participants signed an informed written consent before enrollment.

## 3. Results

Our study included a group of 50 patients matched into two subgroups according to BMI. We assessed demographic, anthropometric, hemodynamic and paraclinical parameters which are presented in Table 1. The mean age of patients with obesity was higher than those in the second group (*p* = 0.021), being a statistically significant parameter in our study. Both groups included predominantly female patients (84% vs. 68%). In terms of anthropometric data, as expected, patients with morbid obesity had associated higher BMI values (43.9 ± 6.07 kg/m^2^ vs. 24.24 ± 3.15 kg/m^2^, *p* = 0.0001), waist circumference (125.5 ± 18.68 cm vs. 83.04 ± 8.75 cm, *p* = 0.0001) and WHR (0.96 ± 0.10 vs. 0.83 ± 0.08, *p* = 0.0001). The central obesity index is another statistically significant parameter in our study, its mean values being 25% higher than those of patients in the second group (0.75 ± 0.08 vs. 0.50 ± 0.06, *p* = 0.0001). In addition, in this category of patients, the presence of obesity was associated with higher mean values of the blood pressure profile, both in SBP (*p* = 0.002) and DBP (*p* = 0.004) or PP (*p* = 0.013).

Dyslipidemia and hyperuricemia were more frequently associated in patients with morbid obesity. Mean serum levels of total cholesterol (201.4 ± 27.17 mg/dl vs. 197.80 ± 41.39 mg/dl, *p* = 0.718), LDL cholesterol (127.68 ± 23.48 mg/dl vs. 125.04 ± 39.97 mg/dl, *p* = 0.76) and serum triglycerides (124.32 ± 17.96 mg/dl vs. 121.24 ± 25.74 mg/dl, *p* = 0.86) were higher in the first group, but without associating statistical significance.

Changes in the carbohydrate profile were more prominent compared to changes in the lipid profile in our study, with statistical analysis highlighting the statistical significance of fasting glucose, insulinemia, insulin sensitivity and insulin resistance as seen in Table 1. Chemerin and adiponectin are the main adipokines assessed in our studied patients. Higher mean serum values were reported in patients with morbid obesity, being statistically significant parameters (18.05 ±1.155 ng/mL vs. 16.36 ± 1.49 ng/mL, *p* = 0.0003 for adiponectin and 12.22 ± 3.80 ng/mL vs. 9.10 ± 1.89 ng/mL, *p* = 0.0001 for chemerin). Besides these, the ratio of the two cytokines also associated significant variations between the two groups (0.67 ± 0.18 vs. 0.55 ± 0.12, *p* = 0.005). Of the inflammatory markers, TNF-α was assessed in patients from both groups, with higher mean serum values in patients from the second group (7.49 ± 3.38 pg/mL vs. 11.34 ± 11.42 pg/mL, *p* = 0.116).

In our study, only SEVR as a parameter of arterial stiffness, was at the limit of statistical significance (*p* = 0.054) between the two studied groups.

Among the echocardiographic parameters evaluated, LVEF (*p* = 0.0073), epicardial adipose tissue thickness (*p* = 0.0001) and LV dimensions (*p* = 0.0001 for end-diastole diameter, *p* = 0.0024 for posterior wall and *p* = 0.0096 for interventricular septum) are statistically significant parameters in our group. The presence of subclinical atherosclerosis was assessed using carotid Doppler ultrasound. Patients with morbid obesity have associated higher mean serum values of carotid intima–media thickness (*p* = 0.0033), diastolic thickness of the media (*p* = 0.0159), relative wall thickness (*p* = 0.0295) and carotid cross-sectional area (*p* = 0.0042).

**Figure 2:**
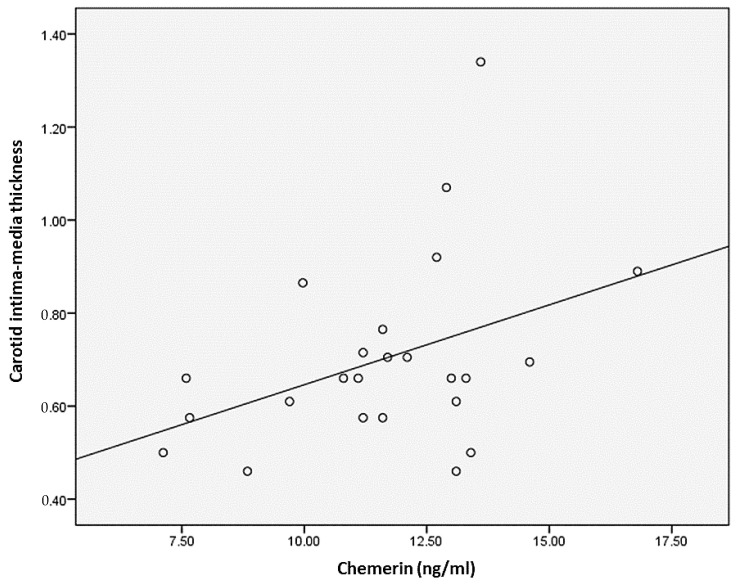
Correlations between serum chemerin levels and carotid intima–media thickness.

We realized a series of correlations between the imaging parameters presented above and the biological or histological ones. In patients with morbid obesity, we identified positive correlations between chemerin and carotid intima–media thickness (*p* = 0.050) (Figure 2), systolic thickness of the media (*p* = 0.015) and diastolic thickness of the media (*p* = 0.001). The same carotid parameters are also statistically significantly correlated with the chemerin/adiponectin ratio, data shown in Table 2.

Table 3 presents the correlations between imaging parameters and adipocyte size or blood vessel wall thickness. Statistical analysis revealed directly proportional relationships between LV mass index (*p* = 0.008), systolic thickness of the media (*p =* 0.009), diastolic thickness of the media (*p* = 0.007), carotid cross-sectional area (*p* = 0.001) and blood vessel wall thickness. Relative wall thickness positively correlates with adipocyte size (*p* = 0.023).

In addition to the descriptive analysis presented above, we performed several multivariate regression models with the dependent variable adipocyte size, carotid intima–media thickness, PWVAo, SEVR and aortic Aix, which are presented in Table 4. Analyzing the obtained data, we can conclude that imaging parameters are predictive factors of arterial stiffness in patients with morbid obesity, which justifies their evaluation as part of diagnostic and therapeutic management.

## 4. Discussion

We conducted a study in which 50 patients with a similar demographic profile (age and gender matched) were evaluated by cardiologist prior to laparoscopic surgery for morbid obesity or other associated benign pathologies. In addition to the analysis of biological parameters, imaging (echocardiographic and carotid Doppler ultrasound) and functional explorations for the assessment of arterial stiffness parameters were performed. Data obtained from patients in the two groups were used for statistical analysis with a focus on identifying predictors associated with arterial stiffness in patients with morbid obesity.

In our study, among echocardiographic parameters, epicardial fat thickness (*p* = 0.0001), LVEF (*p* = 0.0073) and LV dimensions are statistically significant parameters. In the case of epicardial adipose tissue thickness, multivariate statistical analysis demonstrated the role of predictors for adipocyte size which in turn modulates PWVAo, SEVR and aortic Aix. Similar results have been reported by studies in the literature. The investigation of epicardial adipose tissue thickness is one of the modern research directions of recent years in view of its multiple pathophysiological implications in relation to arterial stiffness and the development or progression of atherosclerotic processes that underlie the cardiovascular continuum. Britton et al. [26] highlighted that visceral adiposity has a predictive role for CVD occurrence, improving the associated risk by an additional 16.3% compared to BMI. In a similar clinical study, Mahabadi et al. [27] demonstrated that epicardial adipose tissue thickness relates to the risk of an acute coronary event by 1.54 (both fatal and non-fatal), independent of the presence of classified cardiovascular risk factors. Al-Talabany et al. [28] concluded that there is a direct independent relationship between epicardial adipose tissue thickness and PWV, leading to an increased cardiovascular risk secondary to mediation of the inflammatory processes involved. A recently published meta-analysis revealed that patients with increased visceral adiposity have a 1.26-fold increased risk of associating atherosclerotic plaques with a high risk of complications [29]. These findings also represent future research directions, by developing therapeutic agents specifically targeting epicardial adipose tissue to reduce the risk of an acute cardiovascular event.

Yang et al. [30] analyzed a cohort of 276 adolescents (aged 10 to 20 years old) with obesity and identified that age, insulin resistance, serum total cholesterol or HDL cholesterol and C-reactive protein are independent factors correlated with epicardial adipose tissue thickness. Epicardial adipose tissue thickness correlates with increased LV mass index or relative wall thickness values, thus having a negative impact on cardiac geometry or cardiac function. In our study, patients with morbid obesity had higher LV mass index values compared to patients in the second group (*p* = 0.2851), while mean relative wall thickness values were similar in both groups. (*p* = 0.9702) None of the parameters mentioned above reached the limit of statistical significance.

Epicardial adipose tissue modulates the secretion of adipokines, molecules with an active role in the development of atherosclerosis [7,31,32]. In our study, we did not find statistically significant correlations between serum adipokine levels and epicardial adipose tissue thickness, but we attribute this result to the analysis of a small group of patients. It is also responsible for the production of pro-inflammatory molecules such as TNF-α. Patients in our study with morbid obesity, although associated with higher epicardial adipose tissue thickness, had lower serum TNF-α level than patients in the second group.

The relationship between arterial stiffness and epicardial adipose tissue has been investigated in multiple clinical studies published over the past decade. Epicardial adipose tissue thickness correlates directly proportionally and independently with brachial PWV, and is a useful clinical predictor of subclinical atherosclerotic damage [33]. Pulse pressure, carotid intima–media thickness, SBP and age are other parameters that correlate positively with epicardial fat in obese and hypertensive patients [34].

The relationship between epicardial adipose tissue, LV mass index and carotid intima–media thickness was investigated by Cabrera-Rego et al. [35]. These investigators reported that children and adolescents with obesity have higher epicardial adipose tissue thickness (*p* < 0.001), LV mass (*p* = 0.008), carotid intima–media thickness (*p* = 0.019) and PWV (*p* = 0.007) values. In addition, patients with increased epicardial adipose tissue have a 3.19-fold increased risk of developing subclinical carotid atherosclerosis, correlated with serum adiponectin values [36]. In a similar study, Demir et al. [37] analyzed two groups of patients with and without documented arterial stiffness and demonstrated that PWV correlates with epicardial adipose thickness, age and SBP in patients in the first group. An epicardial adipose tissue thickness over 5.5 mm has a sensitivity of 77% and a specificity of 65% for predicting low arterial compliance in asymptomatic hypertensive patients [38]. In patients with heart failure with preserved LVEF, an epicardial adipose tissue greater than 3.55 mm correlates with increased brachial PWV values, after adjusting for demographic and hemodynamic parameters [39].

In our study, insulin resistance is a statistically significant parameter (*p* = 0.001). The connection between this biochemical parameter and arterial stiffness was demonstrated by Altin et al. [40]. This group of investigators demonstrated that patients with insulin resistance have increased epicardial adipose tissue thickness and carotid intima–media thickness values, but not femoral intima–media thickness, suggesting arterial stiffness first at the carotid level. In addition, age (*p* < 0.001) and insulin resistance (*p* < 0.001) are predictors of carotid intima–media thickness.

Sen et al. [41] highlighted the role of aortic pulse wave velocity as a predictor for the presence of arterial stiffness demonstrating statistically significant correlations between it and aortic strain or aortic distensibility (*p* < 0.001). This parameter also correlates with the maximum and mean carotid intima–media thickness (*p* < 0.001).

Carotid–femoral PWV associates with relative wall thickness (*p* < 0.05) as well as with LV geometry and function [42]. In our study, we demonstrated the positive correlation between relative wall thickness and adipocyte size (*p* = 0.023), as an indirect parameter determinant for arterial stiffness. Liu et al. [43] analyzed the thickness of the mid-thoracic descending aortic in relation to arterial stiffness and concluded that the increase in PWV is associated with decreased distensibility (*p* < 0.001) of the ascending aorta in hypertensive patients. The relative wall thickness can be assimilated to a marker of LV geometry with a potential role in the assessment of systolic and diastolic dysfunction [44].

LV mass index and aortic ring diameter predicts aortic Aix in a multiple linear regression model in our study. Schott et al. [45] recently demonstrated that Aix is associated with left ventricular wall thickness, relative wall thickness and left ventricular mass index as echocardiographic parameters, thus emphasizing the role of echocardiography in predicting arterial stiffness. Arterial stiffness induces diastolic dysfunction, thus being a risk factor for the development of heart failure with preserved ejection fraction [46]. The same group of investigators concluded that male patients with high AIx values are associated with a 3.2-fold increased risk of developing this clinical form of heart failure.

Increased carotid intima–media thickness values correlate with the presence of myocardial dysfunction in patients with obesity, as judged by abnormal longitudinal strain values (*p* ≤ 0.0001) and lower brachial distensibility (*p* < 0.001) [47]. Vascular distensibility and carotid intima–media thickness are predictive for the occurrence of an acute cardiovascular event in patients with cardiovascular risk factors or vascular damage, with increased risk being directly proportional to increased carotid intima–media thickness and decreased arterial distensibility [48]. Increased mean blood pressure, BMI and serum triglyceride levels are determinants of the progression of arterial stiffness as measured by increased carotid intima–media thickness [49]. The predictive value of carotid intima–media thickness is higher for stroke than for myocardial infarction, each 0.1 mm increase being associated with a 1.15-fold increase in risk [50,51].

Carotid media thickness has both prognostic and therapeutic value. In our study, we demonstrated that its systolic and diastolic values correlate with serum adipokine levels in patients with morbid obesity (*p* < 0.05). It also correlates with blood vessel wall thickness (*p* < 0.05). The role of arterial stiffness markers has been demonstrated in multiple studies to date, with emphasis on the predictive value on associated cardiovascular risk [52]. The carotid intima–media thickness assessment is extremely useful in patients with intermediate cardiovascular risk, having a superior prognostic role in association with the Framingham score [53].

Relative wall thickness (*p* = 0.02) and carotid cross-sectional area (*p* = 0.0042) are two imaging parameters calculated using carotid ultrasound with statistical significance in our study. The carotid cross-sectional area correlates positively with blood vessel wall thickness (*p* = 0.001) and is a determinant of adipocyte size that indirectly modulates arterial stiffness parameters in patients with morbid obesity. The role of this parameter as a determinant of carotid arterial stiffness (known as a surrogate of aortic stiffness) has been extensively discussed in the literature. The presence of carotid arterial stiffness correlates with cognitive decline and increases the risk of stroke.

Hypertrophy of the arterial vascular wall induces the asymmetry of distensibility at the carotid level, which explains the varied results of antihypertensive medication reported for the investigated vascular bed [54,55,56]. Boutouyrie et al. [57] have previously highlighted that the LV end-diastolic volume index correlates positively with carotid cross-sectional area (*p* < 0.0001) and compliance (*p* < 0.0001), while the LV wall thickness and mass–volume index is negatively associated with carotid distensibility and compliance (*p* < 0.001). In the clinical study conducted by the same group of investigators, carotid cross-sectional area correlates statistically significantly with LV mass index (*p* < 0.02), thus suggesting the interconnection between morphological and functional changes at the carotid level and LV geometry. The importance of assessing carotid arterial stiffness parameters in patients with obesity was also demonstrated by Ferberg et al. [58] who pointed out that BMI, waist circumference and percentage of body fat are superior predictors for carotid intima–media thickness compared to mean arterial pressure.

Our study has several limitations due to the small number of patients included, as well as the lack of post-operative follow-up of the patients with morbid obesity necessary for assessment of the evolution of arterial stiffness parameters in relation to imaging determinants.

## 5. Conclusions

Patients with morbid obesity show changes in biological and functional parameters (echocardiographic, arteriographic) secondary to excessive adiposity with the associated pro-inflammatory status having a high risk of developing an acute cardiovascular event in the context of early arterial stiffness. The multimodality imaging assessment provides prognostic and therapeutic guidance alike. Our results suggest that epicardial adipose tissue thickness, carotid intima–media thickness, relative wall thickness and carotid cross-sectional area might be useful imaging parameters for the early prediction of arterial stiffness in patients with morbid obesity.

## Figures and Tables

**Table 1:** Demographics, anthropometric and paraclinical parameters included in the statistical analysis.

	Obese Patients (*n* = 25)	Non-Obese Patients (*n* = 25)	*p*
**Demographics, antropometric and hemodynamic parameters**			
Age (y)	39.24 ± 8.74	43.36 ± 13.9	0.021
Female sex (%)	21 (84%)	17 (68%)	-
BMI (kg/m^2^)	43.9 ± 6.07	24.24 ± 3.15	0.0001
Systolic blood pressure (mmHg)	129.36 ± 13.03	118.04 ± 11.72	0.002
Diastolic blood pressure (mmHg)	75.28 ± 11.12	67.08 ± 7.89	0.004
Pulse pressure (mmHg)	59.08 ± 11.28	51.32 ± 9.98	0.013
**Biological parameters**			
Fasting glucose (mg/dL)	99.28 ± 14.62	88.32 ± 8.80	0.002
Insulinemia (µU/mL)	24.47 ± 6.16	8.23 ± 7.98	0.0004
Insulin sensitivity*	6.45 ± 3.73	1.82 ± 1.87	0.0001
Insulin resistance (M/mU/l)	0.13 ± 0.02	0.16 ± 0.12	0.001
Uric acid (mg/dL)	6.79 ± 2.19	5.29 ± 1.48	0.006
**Arterial stiffness parameters**			
Aortic AIx (%)	24.1 ± 12.1	35.1 ± 16.2	0.090
Brachial AIx (%)	−26.7 ± 0.24	−5.5 ± 0.32	0.110
Aortic SBP (mmHg)	128.74 ± 20.81	119.42 ± 20.18	0.114
Aortic PP (mmHg)	52.26 ± 10.76	50.66 ± 12.69	0.633
DRA	49.68 ± 11.38	45.32 ± 18.82	0.321
SAI (%)	48.82 ± 3.81	46.41 ± 6.36	0.112
DAI (%)	51.28 ± 3.80	53.61 ± 6.06	0.111
SEVR	1.06 ± 0.16	1.19 ± 0.28	0.054
PWVAo (m/s)	9.59 ± 2.38	8.92 ± 2.14	0.305
**Perivisceral adipose tissue parameters**			
Adipocyte size (µm)	9.34 ± 2.11	6.62 ± 1.78	0.027
Blood vessel wall thickness (µm)	8.79 ± 2.12	6.92 ± 1.48	0.0001
**Echocardiographic data**			
LV mass index (g/m^2^)	75.10 ± 18.85	70.25 ± 12.16	0.2851
Aortic ring (mm)	17.20 ± 1.50	17.36 ± 3.13	0.7112
LVEF (%)	60.72 ± 5.49	64.40 ± 3.51	0.0073
Epicardial fat thickness (mm)	0.53 ± 0.13	0.32 ± 0.08	0.0001
LV diastole diameter (mm)	50.44 ± 4.38	44.48 ± 3.22	0.0001
LV posterior wall (mm)	9.19 ± 1.48	8.10 ± 0.78	0.0024
Interventricular septum (mm)	10.10 ± 1.59	9.02 ± 1.20	0.0096
Relative wall thickness	0.37 ± 0.06	0.37 ± 0.04	0.9702
**Carotid Doppler Ultrasound**			
Carotid intima–media thickness (mm)	0.72 ± 0.21	0.57 ± 0.07	0.0033
Systolic thickness of the media (mm)	7.19 ± 1.05	6.78 ± 0.58	0.0888
Diastolic thickness of the media (mm)	6.75 ± 0.72	6.27 ± 0.63	0.0159
Relative wall thickness	0.21 ± 0.06	0.18 ± 0.03	0.0295
Wall-to-lumen thickness ratio	0.12 ± 0.04	0.11 ± 0.02	0.0930
Carotid cross-sectional area (mm^2^)	20.05 ± 7.68	14.98 ± 2.82	0.0042

All values are expressed as mean standard deviation (SD) or *n* (%); y: years; BMI: body mass index; WHR: waist to hip ratio; SBP: systolic blood pressure; DBP: diastolic blood pressure; MBP: mean blood pressure; PP: pulse pressure; DRA: diastolic reflection area; SAI: systolic area under the pulse wave curve; DAI: diastolic area under the pulse wave; PWVAo: aortic pulse wave velocity; AIx: augmentation index; SEVR: subendocardial viability index; LV: left ventricle; LVEF: left ventricle ejection fraction; * We used the quantitative insulin sensitivity check index (QUICKI).

**Table 2:** Correlations between chemerin and chemerin/adiponectin ratio with imaging parameters.

	Patients with Morbid Obesity			
Parameters	Chemerin		Chemerin/Adiponectin Ratio	
	r	*p*	r	*p*
**Echocardiographic data**				
LV mass index (g/m^2^)	0.373	0.072	0.319	0.120
Aortic ring (mm)	0.283	0.202	0.365	0.086
LVEF (%)	−0.044	0.839	0.103	0.626
Epicardial adipose tissue thickness (mm)	0.198	0.354	0.030	0.886
**Carotid Doppler Ultrasound**				
Carotid intima–media thickness (mm)	0.404	0.050	0.447	0.025
Systolic thickness of the media (mm)	0.492	0.015	0.480	0.015
Diastolic thickness of the media (mm)	0.620	0.001	0.480	0.015
Relative wall thickness	0.165	0.441	0.286	0.166
Wall-to-lumen thickness ratio	0.106	0.632	0.246	0.247
Carotid cross-sectional area (mm^2^)	0.045	0.834	0.320	0.119

r: Pearson correlation; LV: left ventricle; LVEF: left ventricle ejection fraction.

**Table 3:** Correlations between adipocyte size and blood vessel wall thickness with imaging parameters.

	Non-Obese Patients				Patients with Morbid Obesity			
Parameters	Adipocyte Size		Blood Vessel Wall Thickness		Adipocyte Size		Blood Vessel Wall Thickness	
	r	*p*	r	*p*	r	*p*	r	*p*
**Echocardiographic data**								
LV mass index (g/m^2^)	−0.220	0.123	0.218	0.128	−0.217	0.129	0.380	0.008
Aortic ring (mm)	−0.147	0.336	0.093	0.547	0.132	0.402	0.273	0.084
LVEF (%)	0.234	0.135	−0.135	0.390	0.177	0.241	−0.007	0.962
Epicardial adipose tissue thickness (mm)	−0.14	0.925	0.044	0.761	0.242	0.092	−0.057	0.691
**Carotid Doppler Ultrasound**								
Carotid intima–media thickness	−0.059	0.689	0.167	0.258	0.206	0.158	0.257	0.078
Systolic thickness of the media	−0.104	0.468	0.149	0.303	0.13	0.925	0.372	0.009
Diastolic thickness of the media	−0.132	0.361	−0.007	0.963	−0.060	0.674	0.385	0.007
Relative wall thickness	0.00	0.889	0.050	0.726	0.324	0.023	0.093	0.513
Wall-to-lumen thickness ratio	−0.043	0.761	0.047	0.743	0.210	0.141	0.153	0.283
Carotid cross-sectional area	−0.130	0.362	0.047	0.743	0.023	0.870	0.473	0.001

r: Pearson correlation; LV: left ventricle; LVEF: left ventricle ejection fraction.

**Table 4:** Multiple linear regression models including parameters associated with arterial stiffness.

Dependent Variable	Independent Variable	Coefficient	*p*-Value	*p*-Value Model	R^2^ Adjusted
**Adipocyte size**	Constant	−2.021	0.543	0.0001	0.73
	Chemerine/adiponectine ratio	0.379	0.858		
	Triglycerides	0.015	0.001		
	SEVR	6.198	0.001		
	Carotid cross-sectional area	0.085	0.025		
	Epicardial fat thickness	6.724	0.008		
**Carotid intima–media thickness**	Constant	−0.691	0.007	0.0001	0.61
	Adipocyte size	0.035	0.014		
	Blood vessel wall thickness	0.007	0.645		
	Diastolic thickness of the media	0.09	0.007		
	Wall-to-lumen thickness ratio	2.995	0.001		
**PWVAo**	Constant	5.712	0.032	0.0001	
	Adipocyte size	0.065	0.422		
	Blood vessel wall thickness	0.077	0.294		
	Waist circumference	0.063	0.001		0.91
	White blood cells	0	0.003		
	TNF-α	0.11	0.042		
**SEVR**	Constant	2.985	0.000001	0.0001	0.99
	Adipocyte size	0.003	0.052		
	Blood vessel wall thickness	−0.002	0.115		
	Waist circumference	0.001	0.043		
	Basophils	0.062	0.003		
	Serum fibrinogen	0	0.023		
	Serum creatinine	0.077	0.011		
	Insulin sensitivity	0.687	0.009		
	SAI	−0.043	0.000001		
**Aortic Aix**	Constant	65.554	0.009	0.0001	0.85
	Adipocyte size	1.653	0.011		
	Blood vessel wall thickness	−0.547	0.415		
	HDL cholesterol	0.398	0.016		
	Heart rate	−0.542	0.003		
	DRA	−0.569	0.000001		
	LV mass index	0.503	0.000001		
	Ao ring dimmensions	−2.377	0.007		

SAI: systolic area under the pulse wave curve; PWVAo: aortic pulse wave velocity; SEVR: subendocardial viability index; AIx: augmentation index; TNF: tumor necrosis factor; HDL: high-density lipoproteins; LV: left ventricle; Ao: aortic.

## Data Availability

The data presented in this study are available on request from the corresponding author. The data are not publicly available due to local policies.
